# Vector polymorphic beam

**DOI:** 10.1038/s41598-018-26126-9

**Published:** 2018-05-16

**Authors:** José A. Rodrigo, Tatiana Alieva

**Affiliations:** Universidad Complutense de Madrid, Facultad de Ciencias Físicas, Ciudad Universitaria s/n, Madrid, 28040 Spain

## Abstract

A scalar polymorphic beam is designed with independent control of its intensity and phase along a strongly focused laser curve of arbitrary shape. This kind of beam has been found crucial in the creation of freestyle laser traps able to confine and drive the motion of micro/nano-particles along reconfigurable 3D trajectories in real time. Here, we present and experimentally prove the concept of vector polymorphic beam adding the benefit of independent design of the light polarization along arbitrary curves. In particular, we consider polarization shaped tangential and orthogonal to the curve that are of high interest in optical manipulation and laser micromachining. The vector polymorphic beam is described by a surprisingly simple closed-form expression and can be easily generated by using a computer generated hologram.

## Introduction

Shaping a coherent laser beam with independent control of its intensity, phase and polarization is a long posted problem of high interest in science and technology. This is particularly important in areas such as optical manipulation of micro/nano-particles and light material processing, where laser beams strongly focused in the form of diffraction-limited patterns such as points and curves are required. For example, it is known that the three-dimensional (3D) high intensity gradients of a focused laser beam yield optical forces responsible for stable 3D trapping of particles^1^. The phase gradients of the beam can be also exploited to exert optical forces able to drive the motion of the particles along different trajectories^2–5^. The combined use of high intensity and phase gradients allows for improving laser micromachining tools^6,7^. It has been also reported that vector Gaussian beams with radial and/or tangential (azimuthal) polarization have advantages for laser material processing (micromachining such as drilling and cutting), e.g.: tangential polarization provides increased laser micro-drilling velocities and generation of thin capillaries with high aspect ratios in thick sheets^8,9^. Moreover, it has been demonstrated that femtosecond vortex pulses tightly focused onto the surface of dielectric media allows creating subwavelength ripples whose orientation depends on the polarization direction^10,11^. The ability to change the polarization state in the focal plane by tuning the vortex topological charge^11^ adds new income in the development of such micro- and nano-scale surface structuring.

**Figure 1:**
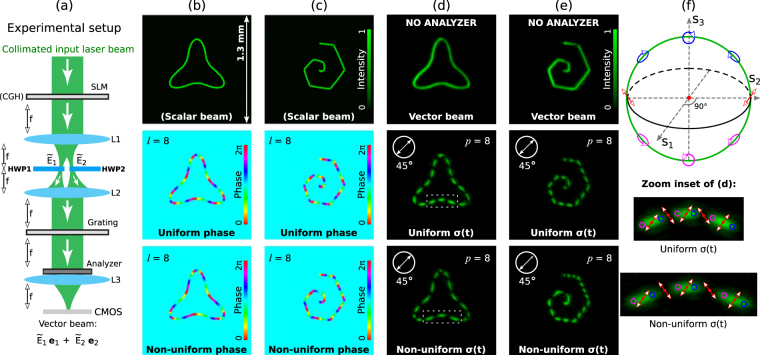
(**a**) Sketch of the system used for the generation and analysis of vector beams. (**b**) Intensity and phase distributions of a scalar polymorphic beam shaped in form of triangular-like curve given by Eq. (9) with parameters **q** = (1, 1.8, 1, 2, 1.4, 6) and *ρ*(*t*) = constant. The second and third rows show the uniform and non-uniform phase distributions (charge *l* = 8) prescribed along the curve. (**c**) Intensity and phase shaped along a spiral curve with **q** = (1, 1, 250, 100, 100, 6) and *ρ*(*t*) ∝ *t*. (**d**,**e**) Experimental results: vector polymorphic beam with uniform and non-uniform polarization variation, ${\bf{e}}(t)=({e}^{{\rm{i}}2p\pi \sigma (t)},\,{e}^{-{\rm{i}}2p\pi \sigma (t)})/\sqrt{2}$, prescribed along the curve. In the experiments the focal length is f = 150 mm and the input collimated laser beam (linear polarized) has a wavelength of *λ* = 532 nm. (**f**) Changes of the polarization state along the curved vector beams considered in this example. The repetitive changes of the polarization, associated with the movements on the green-marked meridians of the Poincaré sphere, along the curves are illustrated in the corresponding zoom inserts of (**d**).

In general, a vector beam **E**(*x*, *y*) = *A*(*x*, *y*)**e** with arbitrary complex field amplitude *A*(*x*, *y*) and polarization distribution **e** = (*a*_1_(*x*, *y*), *a*_2_(*x*, *y*)), at a given transverse *xy*-plane, can be generated by using computer generated holograms (CGHs) addressed into programmable spatial light modulators (SLMs) as reported elsewhere^12–16^. Here **e** is a Jones vector where *a*_1,2_(*x*, *y*) are complex functions such that |*a*_1_(*x*, *y*)|^2^ + |*a*_2_(*x*, *y*)|^2^ = 1. Indeed, a combination of two collinear beams **E**_1_(*x*, *y*) = *A*(*x*, *y*)*a*_1_(*x*, *y*)**e**_1_ and **E**_2_(*x*, *y*) = *A*(*x*, *y*)*a*_2_(*x*, *y*)**e**_2_ with orthogonal linear polarization states **e**_1_ = (1, 0) and **e**_2_ = (0, 1) forms a vector beam. Circular polarized beams ${{\bf{e}}}_{\mathrm{1,2}}^{c}=(1,\pm \,i)$ can be also used for this task. The superposition of the beams **E**_1_ and **E**_2_ created by using two SLMs is often achieved applying a Mach-Zehnder interferometer^16^ or a common-path interferometer with a Ronchi grating^17^ as the one sketched in Fig. 1(a). Numerous vector beams have been created by using this approach^12–16^, however, the challenging problem of the design and generation vector beams strongly focused in form of a diffraction-limited light curve of arbitrary shape with independent control of its intensity, phase and polarization distributions along it has not been completely solved. While in^18^, an inverse design based on a numerical calculation procedure has been proposed for complete 2D shaping of the optical focal field with the prescribed distribution of intensity, phase and polarization, however, an analytical expression for the required beam simplifying the light curve generating process has not been found. On the other hand in^19^, using the expression for scalar curved beams proposed by us in^20^, a particular case of polarization shaping of curved vector beams in three dimensions has been demonstrated. Note that the approach reported in^19^ does not allow shaping an arbitrary phase along the curve.

In this work we present a kind of vector beam, referred to as vector polymorphic beam, that solves this challenging problem of beam shaping. It is based on its scalar analogous beam^21^, which can be strongly focused in form of diffraction-limited light curve of arbitrary shape with the following key properties: high 3D intensity gradients, independent design of the intensity and phase distributions (both of them can be arbitrary) along the curve according to the considered application. The scalar polymorphic beam has been used to create freestyle laser traps providing both optical confinement and transport of micro/nano-particles along reconfigurable trajectories^5,20,22^. Apart from optical manipulation, scalar polymorphic beams have been also applied for laser printing of plasmonic nanoparticles^23^ illustrating their possible application for single-shot laser lithography, micro-machining (drilling and marking)^7–9^, etc. Its design has an inherent versatility that can be exploited to create other types of coherent beams such as electron beams shaped along curves, as it has been shown in^24^. Another advantage is that this kind of beam shaping technique does not require the use of iterative algorithms, thus enabling direct and fast generation of such laser curves. Moreover, it can be easily implemented by using a CGH addressed into a conventional programmable SLM.

In next section we propose a simple technique for the generation of vector polymorphic beams with tailored polarization distribution along the light curve, thus enlarging the practical applications of their scalar counterparts. In section 3 the technique is experimentally verified. The article ends with conclusion remarks.

## Principle of the Technique

The vector polymorphic beam is defined by:

1 $$\begin{array}{rcl}{\bf{E}}(x,y) & = & {E}_{1}(x,y){{\bf{e}}}_{1}+{E}_{2}(x,y){{\bf{e}}}_{2}\\  & = & {\int }_{0}^{T}({g}_{1}(t){{\bf{e}}}_{1}+{g}_{2}(t){{\bf{e}}}_{2})\,\exp \,[-\,{\rm{i}}\frac{k}{{\rm{f}}}R(t)(x\,\cos \,t+y\,\sin \,t)]\,{\rm{d}}t,\end{array}$$

with **e**_1,2_ corresponding to orthogonal polarization components, for example, linear **e**_1_ = (1, 0) and **e**_2_ = (0, 1) or circular ${{{\bf{e}}}^{{\rm{c}}}}_{\mathrm{1,2}}$ = (1, ±i) ones. Specifically, *g*_1,2_(*t*) = *g*(*t*)*a*_1,2_(*t*) are weight functions defining the intensity and phase distributions along the curve, while *a*_1,2_(*t*) controls the polarization along it assuming |*a*_1_(*t*)|^2^ + |*a*_2_(*t*)|^2^ = 1. Here, *R*(*t*) is the radius of an arbitrary 2D curve given in polar coordinates that can be either closed (*T* = 2*π*) or open, f is a normalization constant, *T* stands for the maximum value of the azimuthal angle *t*, while *k* = 2*π/λ* with *λ* being the light wavelength.

Let us first briefly recall the main characteristics of the scalar polymorphic beam^21^

2 $$E(x,y)={\int }_{0}^{T}g(t)\,\exp \,[-\,{\rm{i}}\frac{k}{{\rm{f}}}R(t)(x\,\cos \,t+y\,\sin \,t)]\,{\rm{d}}t,$$

used in the design of the vector polymorphic beam Eq. (1). To create the light curve, the polymorphic beam is Fourier transformed^21^:

3 $$\begin{array}{rcl}\tilde{E}(u,v) & = & \frac{1}{{\rm{i}}\lambda {\rm{f}}}\int E(x,y)\,\exp \,[-\,{\rm{i}}\frac{k}{{\rm{f}}}(xu+yv)]\,{\rm{d}}x{\rm{d}}y\\  & = & \frac{\lambda {\rm{f}}}{{\rm{i}}}{\int }_{0}^{T}g(t)\delta (u+R(t)\,\cos \,t)\delta (v+R(t)\,\sin \,t){\rm{d}}t,\end{array}$$

by using a convergent lens of focal length f. Thus, the shape of the beam $\tilde{E}(u,v)$ in the focal plane is described by the 2D curve written in parametric form as **c**(*t*) = (*u*(*t*), *v*(*t*)), with *u*(*t*) = −*R*(*t*) cos *t* and *v*(*t*) = −*R*(*t*) sin *t*. While, the complex weight function

4 $$g(t)=|g(t)|\,\exp \,[{\rm{i}}\frac{2\pi l}{S(T)}S(t)],$$

controls the amplitude and phase distributions along the curve. Specifically, the field amplitude distribution along the curve is given by $|\tilde{E}(u(t),\,v(t))|=|g(t)|/\kappa |{\bf{c}}^{\prime} (t)|$, where: $|{\bf{c}}^{\prime} (t)|=\sqrt{R^{\prime} {(t)}^{2}+R{(t)}^{2}}$ with **c**′(*t*) = d**c**(*t*)/d*t*, and *κ* = *L*/*λ*f with $L={\int }_{0}^{T}|{\bf{c}}^{\prime} (\tau )|{\rm{d}}\tau$ being the curve length^21^. While, the phase of *g*(*t*) is controlled by the real function *S*(*t*) describing the phase variation along the curve. Note that the parameter *l* defines the phase accumulation along the entire curve^21^. For closed curves the phase accumulation is 2*πl* and *l* corresponds to the vortex topological charge^25^. For instance, a light curve with uniform intensity is obtained by using |*g*(*t*)| = *E*_0_*κ*|**c**′(*t*)| (with dimension of electric field) while

5 $$S(t)={\int }_{0}^{t}|{\bf{c}}^{\prime} (\tau )|{\rm{d}}\tau ,$$

sets a uniform phase distribution along the curve **c**(*t*). A non uniform phase shaped along the curve can be easily obtained by using, for example, the following constraint

6 $$S(t)={\int }_{0}^{t}{R}^{\alpha }(\tau ){\rm{d}}\tau ,$$

with *α* being a real number^21^. For instance, in Fig. 1(b) it is shown a scalar polymorphic beam focused in form of triangular-like curve with a topological charge *l* = 8, for the case of uniform [Eq. (5)] and non-uniform [Eq. (6) with *α* = 2] phase distribution. Note that the change of either the phase distribution and/or value of *l* does not alter the shape and size of the beam curve, independently whether the curve is closed or open as the spiral one demonstrated in Fig. 1(c).

Such a versatile control of amplitude and phase along the curve results crucial for creating vector polymorphic beams. For example, to adapt the polarization **e**(*t*) = (*a*_1_(*t*), *a*_2_(*t*)) to the curve shape, the phase of *a*_1,2_(*t*) can be expressed similar to Eq. (4) as it follows: exp[i2*πp*_1,2_*σ*_1,2_(*t*)] with *σ*_1,2_(*t*) = *S*_1,2_(*t*)/*S*_1,2_(*T*) and *p*_1,2_ being real numbers. Note that *σ*_1,2_(*t*) ∈ [0, 1] and the variation of the Jones vector along the curve can be also uniform if *S*_1,2_(*t*) is described by Eq. (5), or non-uniform when it is described for example by Eq. (6). Thus, when $|{a}_{1}(t)|=|{a}_{2}(t)|=\mathrm{1/}\sqrt{2}$, *σ*_1,2_(*t*) = *σ*(*t*) and *p*_2_ = −*p*_1_ = *p* the polarization defined by the Jones vector ${\bf{e}}(t)=({e}^{{\rm{i}}2\pi p\sigma (t)},\,{e}^{-{\rm{i}}2\pi p\sigma (t)})/\sqrt{2}$ performs a 2*p* rotation along two meridians of the polarization Poincaré sphere with an azimuthal angle distant 180° between each other. In this case the global phase of the vector beam is given by the phase of *g*(*t*), Eq. (4). Other combinations of *a*_1,2_(*t*), allows for more complex movements along the polarization sphere.

A polarization tangential to the curve results more relevant in practical applications such as laser material processing and micro-machining. Indeed, as pointed out in^8,9^ tangential polarization yields improved laser drilling on materials. Phase gradients of scalar vortex beams have been also proved successful for clearer and smoother processed surfaces^6^. Therefore, a vector polymorphic beam with both polarization and phase gradient tangential to the curve opens up promising perspectives. In this case the vector polymorphic beam has to be created with

7 $$\begin{array}{rcl}{a}_{1}(t) & = & (\,-\,R(t)\,\sin \,t+R^{\prime} (t)\,\cos \,t){|{\bf{c}}^{\prime} (t)|}^{-1},\\ {a}_{2}(t) & = & (R^{\prime} (t)\,\sin \,t+R(t)\,\cos \,t){|{\bf{c}}^{\prime} (t)|}^{-1},\end{array}$$

when using orthogonal linear polarization components. Indeed, the Jones vector **e**(*t*) = (*a*_1_(*t*), *a*_2_(*t*)) is tangential to the curve ***c***(*t*). We recall that the weight functions are *g*_1,2_(*t*) = *g*(*t*)*a*_1,2_(*t*) and therefore it is possible obtain the tangential polarization independently of the intensity and phase prescribed by *g*(*t*) along the curve. To set the polarization orthogonal to the curve, *a*_1_(*t*) and *a*_2_(*t*) given by Eq. (7) have to be exchanged.

As we have previously mentioned, orthogonal left- and right-hand circular polarization components ${{\bf{e}}}_{\mathrm{1,2}}^{c}$ = (1, ±i) can be also used to generate a vector polymorphic beam. In this case, in order to create a polarization tangential to the curve, the required functions for two orthogonal components of the beam are defined by

8 $$\begin{array}{rcl}{a}_{1}^{c}(t) & = & \exp \,[-\,{\rm{i}}(\arctan \,(\frac{R(t)}{R^{\prime} (t)})+t)],\\ {a}_{2}^{c}(t) & = & \exp \,[{\rm{i}}(\arctan \,(\frac{R(t)}{R^{\prime} (t)})+t)].\end{array}$$

## Experimental Results

The experimental setup sketched in Fig. 1(a) has been used to generate the vector beams Eq. (1) considered here. It consists of a programmable SLM (Holoeye PLUTO, pixel size of 8 *μ*m) in which a phase-only CGH encoding the beam components as *E*_1_(*x*, *y*) exp (i2*πx*/Λ) + *E*_2_(*x*, *y*) exp (−i2*πx*/Λ) has been addressed by using the approach reported in^26^. We recall that the weight functions are *g*_1,2_(*t*) = *g*(*t*)*a*_1,2_(*t*) and therefore the polarization information of *a*_1,2_(*t*) has been also included into the CGH encoding *E*_1,2_(*x*, *y*). This CGH allows for generating the beams ${\tilde{E}}_{\mathrm{1,2}}(u,v)$ spatially separated at the focal plane of the convergent lens *L*1, where they are respectively modulated by two half-wave plates (HWP1 and HWP2) in order to obtain the required orthogonal linear polarization components, see Fig. 1(a). If orthogonal left- and right-hand circular polarization components are used to generate a vector polymorphic beam then the HWPs of the setup have to be replaced by quarter-wave plates. The beam components ${\tilde{E}}_{\mathrm{1,2}}(u,v)$ are combined by using another convergent lens *L*2 (in our case *L*1 and *L*2 are identical, working together as a 4f system) and a diffraction grating of period Λ (in our case a Ronchi Ruling grating of 20 lp/mm, Edmund Optics). Thus, the focused vector polymorphic beam ${\tilde{E}}_{1}(u,v){{\bf{e}}}_{1}+{\tilde{E}}_{2}(u,v){{\bf{e}}}_{2}$ (the laser curve) is obtained at the focal plane of the lens *L*3, where its intensity distribution has been recorded by a digital camera (color CMOS, Thorlabs, pixel size of 4.7 *μ*m). In our case the analyzer has been set into a programmable rotation stage (Newport URS100BCC).

In the considered examples, the curve is described by

9 $$R(t)=\rho (t)\,{[{|\frac{1}{a}\cos (\frac{m}{4}t)|}^{{n}_{2}}+{|\frac{1}{b}\sin (\frac{m}{4}t)|}^{{n}_{3}}]}^{-\mathrm{1/}{n}_{1}},$$

known as Superformula^27^, that allows for straightforward generation of a large variety of shapes where the real numbers in **q** = (*a*, *b*, *n*_1_, *n*_2_, *n*_3_, *m*) are the design parameters of the curve and *ρ*(*t*) is a non-periodic function of *t* required for the construction of asymmetric and spiral-like curves (e.g.: *ρ*(*t*) ∝ *e*^*βt*^ or *ρ*(*t*) ∝ *t*^*β*^). For example, with **q** = (1, 1, 1, 1, 1, 0) and constant *ρ*(*t*) = *ρ*_0_ a circle of radius *R*(*t*) = *ρ*_0_ is obtained, while for other values of **q** a variety of closed polygons of different symmetry are easily generated^21^.

Let us first consider the experimental examples displayed in Fig. 1(d,e) corresponding to a triangular-like and spiral curves. In this case the vector polymorphic beam focuses into the curve with uniform intensity distribution and constant phase, while its polarization ${\bf{e}}(t)=({e}^{{\rm{i}}2\pi p\sigma (t)},\,{e}^{-{\rm{i}}2\pi p\sigma (t)})/\sqrt{2}$ varies along the curve following the meridian path on the polarization Poincaré sphere as shown in Fig. 1(f). Specifically, Fig. 1(d) shows the experimental results obtained for the triangular-like curve with *l* = 0, *p*_2_ = −*p*_1_ = *p* = 8 and *σ*(*t*) yielding uniform [Eq. (5)] and non-uniform [Eq. (6) with *α* = 2] variation of the polarization along it, see second and third rows respectively. Note that in this case the analyzer has been set at 45° and therefore the measured intensity distribution shows 2*p* = 16 fringes distributed along the curve. The same polarization configurations have been prescribed in the case of the spiral curve, see Fig. 1(e). We underline that in the case of uniform variation of *σ*(*t*) the polarization distribution periodically changes along the curve (*t* ∈ [0, *T*]): it is linearly polarized at 45° and −45° in the points where *σ*(*t*) = *n*/16 and *σ*(*t*) = 1/32 + *n*/16, correspondingly (with *n* = 0, 1, 2, ..., 15). While right(left)-hand circular polarization is obtained in the points where *σ*(*t*) = 1/64 + *n*/16 (and *σ*(*t*) = 3/64 + *n*/16), see also Fig. 1(f). In the case of non-uniform variation of *σ*(*t*) [described by Eq. (6)] this transformation in the polarization is accelerated along the curve.

**Figure 2:**
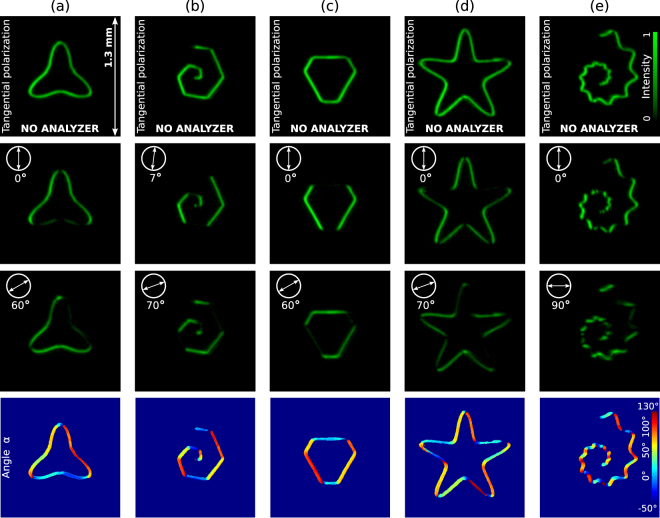
Experimental results: intensity distributions of a vector polymorphic beam ${\tilde{E}}_{1}(u,v){{\bf{e}}}_{1}+{\tilde{E}}_{2}(u,v){{\bf{e}}}_{2}$ with a polarization set tangential to the curve. In this case an uniform phase distribution (*l* = 8), **e**_1_ = (1, 0) and **e**_2_ = (0, 1) have been used. The first row displays the intensity distribution of the generated vector beams while the second and third ones shown their intensities when an analyzer is rotated, see also Supplementary Video 1. The last row shows that the polarization is linear in all the points of the curve and forms an angle *θ* = (arctan(*S*_2_/*S*_1_))/2 with horizontal axis, where *S*_1,2_ are measured Stokes parameters. The polarization is tangential to the curve as expected. Curve parameters: (**a**) triangular-like curve **q** = (1, 1.8, 1, 2, 1.4, 6), (**b**) spiral **q** = (1, 1, 250, 100, 100, 6) with *ρ*(*t*) ∝ *t*, (**c**) a polygon with **q** = (2.7, 2.6, 6, 12, 8.3, 5.3), starfish curve **q** = (10, 10, 2, 7, 7, 5), spiral with **q** = (1, 1, 5, 5, 5, 10) and *ρ*(*t*) ∝ *t*^0.2^.

The examples considered in Fig. 1 have mostly fundamental character. Now we turn to practically important cases: Polymorphic beams with both uniform intensity and phase distribution and linear polarization tangential to the curve. In Fig. 2 and Supplementary Video 1, the rotation of the analyzer indicates that the polarization has been set tangential to the curve as it follows from the further analysis of the Stokes components. Indeed, by calculating the first three Stokes parameters *S*_0_ = *I*(0°) + *I*(90°), *S*_1_ = *I*(0°) − *I*(90°), and *S*_2_ = *I*(45°) − *I*(135°), −where *I*(*θ*) stands for the measured intensity distribution when the analyzer is set at an angle *θ* with respect the horizontal axis − one derives that ${S}_{0}^{2}={S}_{1}^{2}+{S}_{2}^{2}$ and therefore the fourth parameter is *S*_3_ = 0. It means that the polarization is linear in all the points of the curve and forms an angle *θ* = (arctan(*S*_2_/*S*_1_))/2 with horizontal axis as it is shown in the last row of Fig. 2. Note that an uniform phase distribution, given by the function *S*(*t*) in Eq. (5) with *l* = 8, has been used in all the beams displayed in Fig. 2. For example, the uniform phase distribution prescribed in Fig. 2(a,b) is the same as the one displayed in Fig. 1(b,c), respectively.

## Discussions

The vector polymorphic beam can be easily created by using a hologram and provides a direct (non-iterative) way for confining light in the form of a diffraction-limited 2D curve of arbitrary shape and size, with independent control of the intensity, phase and polarization distributions. These degrees of freedom are demanded by relevant applications as for example laser material processing^7–9,23^ and optical manipulation of micro/nano-particles^5,20,22^. Here, the versatility in the design and generation of the vector polymorphic beams have been illustrated in several examples using a straightforward experimental setup. More sophisticated setups, as the ones reported in^9^, can be applied instead for practical implementations of the polymorphic beams. Industrial applications of the polymorphic beams could require a diffraction optical element instead of a liquid crystal SLM for hologram encoding.

## Electronic supplementary material

vector polymorphic beams whose polarization has been set tangential to curves

Legend for supporting videos
