# Supplementary material for: Vector polymorphic beam
Source: Sci Rep. 2018 May 16;8:7698. doi: 10.1038/s41598-018-26126-9 (PMC5955977; doi:10.1038/s41598-018-26126-9)
Supplement: Supplementary file 2 — Legend for supporting videos [file 41598_2018_26126_MOESM2_ESM.pdf]

Vector polymorphic beam

José A. Rodrigo* and Tatiana Alieva

Universidad Complutense de Madrid, Facultad de Ciencias Físicas, Ciudad Universitaria s/n, Madrid 28040, Spain

*Corresponding author: jarmar@fis.ucm.es

The following video is available as supporting information:

Video 1. Experimental results: A rotating analyzer has been used to show vector polymorphic beams whose polarization has been set tangential to different curves corresponding with Fig.2.
